# DiaSurg 2 trial - surgical *vs*. medical treatment of insulin-dependent type 2 diabetes mellitus in patients with a body mass index between 26 and 35 kg/m^2^: study protocol of a randomized controlled multicenter trial - DRKS00004550

**DOI:** 10.1186/1745-6215-14-183

**Published:** 2013-06-20

**Authors:** Hannes G Kenngott, Gabriella Clemens, Matthias Gondan, Jonas Senft, Markus K Diener, Gottfried Rudofsky, Peter P Nawroth, Markus W Büchler, Lars Fischer, Beat P Müller-Stich

**Affiliations:** 1 Department of General, Visceral and Transplantation Surgery, University of Heidelberg, Im Neuenheimer Feld 110, 69120 Heidelberg, Germany; 2 Institute of Medical Biometry and Informatics, University of Heidelberg, Im Neuenheimer Feld 110, 69120 Heidelberg, Germany; 3 Study Center of the German Surgical Society (SDGC), University of Heidelberg, Im Neuenheimer Feld 110, 69120 Heidelberg, Germany; 4 Department of Medicine I and Clinical Chemistry, University of Heidelberg, Im Neuenheimer Feld 110, 69120 Heidelberg, Germany

## Abstract

**Background:**

Type 2 diabetes mellitus (T2DM) is a disease with high prevalence, associated with severe co-morbidities as well as being a huge burden on public health. It is known that glycemic control decreases long-term morbidity and mortality. The current standard therapy for T2DM is medical treatment. Several randomized controlled trials (RCTs) performed in obese patients showed remission of T2DM after bariatric surgery. Recent RCTs have shown bariatric procedures to produce a similar effect in non-morbidly and non-severely obese, insulin-dependent T2DM patients suggesting procedures currently used in bariatric surgery as new therapeutical approach in patients with T2DM. This study aims at investigating whether Roux-en-Y gastric bypass (RYGB) is an efficient treatment for non-severely obese T2DM patients in terms of preventing long-term complications and mortality.

**Methods:**

The DiaSurg 2 trial is a multicenter, open randomized controlled trial comparing RYGB including standardized medical treatment if needed to exclusive standardized medical treatment of T2DM (control group). The primary endpoint is a composite time-to-event endpoint (cardiovascular death, myocardial infarction, coronary bypass, percutaneous coronary intervention, non-fatal stroke, amputation, surgery for peripheral atherosclerotic artery disease), with a follow-up period of 8 years. Insulin-dependent T2DM patients aged between 30 and 65 years will be included and randomly assigned to one of the two groups. The experimental group will receive RYGB and, if needed, standardized medical care, whereas the control group will receive exclusive standardized medical care, both according to the national treatment guidelines for T2DM. Statistical analysis is based on Cox proportional hazards regression for the intention-to-treat population. Assuming a loss to follow-up rate of 20%, 200 patients will be randomly allocated to the comparison groups. A total sample size of *n* = 400 is sufficient to ensure 80% power in a two-tailed significance test at alpha = 5%.

**Discussion:**

The DiaSurg2 trial will yield long-term data (8 years) on diabetes-associated morbidity and mortality in patients with insulin-dependent T2DM receiving either RYGB or standardized medical care.

**Trial registration:**

The trial protocol has been registered in the German Clinical Trials Register DRKS00004550.

## Background

### Rationale of the trial

According to WHO data, in 2000 the worldwide prevalence of type 2 diabetes mellitus (T2DM) was 2.8%, and in 2030 it is expected to rise to 4.4% [1,2]. Approximately 6 million people in Germany are currently suffering from T2DM, among whom 2 million are insulin-dependent [3,4]. A high proportion of these patients already suffer from T2DM-related diseases, such as micro- and/or macrovascular diseases resulting in neuro-, nephro-, retinopathy, myocardial infarction, and stroke [5,6]. The treatment of these co-morbidities renders T2DM one of the most expensive diseases in terms of public health expenditure in Germany [7]. Current guidelines recommend medical therapy for the treatment of T2DM as the gold standard [8,9]. It is known that sufficient glycemic control, defined by the German Diabetes Association and International Diabetes Foundation as HbA1c <6.5%, decreases long-term morbidity [6,8-10]. Current guidelines with the aim of lowering HbA1c-levels are therefore being reconsidered, owing to the growing number of studies showing an increased mortality rate for patients with lower HbA1c [11-14].

Obesity is considered a risk factor for the development of T2DM [15]. For patients with class 2 obesity (BMI >35 kg/m^2^) with co-morbidities and patients with class 3 obesity (BMI >40 kg/m^2^), bariatric surgery is recommended when conservative attempts do not result in substantial weight loss [16]. Existing evidence reports that bariatric surgery not only leads to substantial weight loss, but also to T2DM remission in 42% to 78% of the patients who have undergone RYGB [17-19]. In addition, T2DM-related diseases, such as cardiovascular diseases, nephropathy, retinopathy, neuropathy, hyperlipidemia, and hypertension, are supposed to be avoided or at least delayed in their progress after metabolic surgery [19-21]. In the Swedish Obese Subjects (SOS)-Study 35% of T2DM patients and 19% of patients suffering from preoperative hypertension were still in remission 10 years after surgery [22]. The beneficial effects of bariatric surgery on T2DM appeared shortly after surgery, and before major weight loss, leading to the hypothesis that weight loss alone is not solely responsible for T2DM remission. In an experimental study, Rubino *et al.* were able to prove that these effects are not dependent on weight loss alone [23]. These findings led surgeons to perform bariatric procedures on non-severely obese patients (overweight and obesity class 1; BMI 25–35 kg/m^2^) suffering from T2DM [21,24-26]. A recent randomized controlled, single-center trial in patients with a BMI of 25–35 kg/m^2^ showed T2DM remission in 93% of patients following Roux-en-Y gastric bypass (RYGB) and 47% of patients following laparoscopic gastric sleeve resection [27]. The effect of T2DM remission could therefore also be shown in non-severely obese patients.

This randomized controlled multicenter trial assesses whether bariatric surgery can be used as an alternative in the primary care of T2DM, potentially leading to fewer strokes or cardiovascular death and thus preventing long-term morbidity and mortality well-known in T2DM patients.

### Objective

The aim of DiaSurg 2 is to investigate the time-to-event of T2DM-induced morbidity and mortality after RYGB compared to medical treatment according to the most current clinical guidelines in patients with insulin-dependent T2DM.

### Trial locations

The DiaSurg 2 trial will be conducted in 13 German centers which have expertise in bariatric surgery. So far, the following German centers have already been defined: University of Berlin, University of Dresden, University of Heidelberg, University of Kiel, University of Lübeck, Vivantes Klinikum Spandau Berlin, St.-Martinus Krankenhaus Düsseldorf, Nordwestkrankenhaus Frankfurt am Main, Krankenhaus Sachsenhausen Frankfurt am Main, Klinikum Gera, Wolfart Klinik Gräfelfing, Klinikum Karlsruhe and Klinikum Memmingen. Additional recruitment centers might participate in the study.

## Methods/design

### Trial design

Diasurg 2 trial is a multicenter, open randomized controlled trial. Patients of the experimental group receive RYGB surgery and standardized medical treatment if needed. Patients in the control group receive exclusive standardized medical treatment of T2DM.

### Sample size

Two hundred patients per group (including the expected dropouts) will be randomized for this trial, accounting for a total of 400 patients.

### Patient selection criteria

Eligible patients need to have a diagnosis of insulin-dependent T2DM going back at least 3 months. Furthermore, they should have proof of at least one microvascular manifestation of T2DM (for example, nephropathy, retinopathy, neuropathy) and have sufficient residual endocrine pancreatic function, which is the premise for autogenic glycemic control. Residual endocrine pancreatic function is assessed by glucagon-stimulated fasting C-peptide laboratory tests with a minimum of 1.5 ng/mL. Eligible participants must have a body mass index (BMI) between 26 and 35 kg/m^2^, be aged between 30 and 65 years and be able to provide informed consent. Negative islet cell autoantibody testing will be required for patients who started insulin therapy within 1 year after T2DM diagnosis to eliminate the risk of including patients with autoimmune components (that is, type 1 diabetes mellitus (T1DM) or latent autoimmune diabetes of adults (LADA)). Inclusion and exclusion criteria are depicted in Table 1.

**Table 1:** Inclusion and exclusion criteria

**Inclusion criteria**	**Exclusion criteria**
Diagnosis of T2DM with insulin therapy for at least 3 months and an HbA1c of ≥7%; negative islet cell autoantibody testing will be required for patients that received insulin therapy within 1 year after T2DM diagnosis	Type I diabetes mellitus or latent autoimmune diabetes of adults (LADA)
Proof of at least one microvascular manifestation of diabetes (for example, nephropathy, retinopathy, neuropathy)	T2DM on diet and/or oral medication
Residual pancreatic function, which is the premise for autogenic glycemic control assessed by stimulated fasting C-peptide laboratory tests with a minimum of 1.5 ng/mL	Heart failure (NYHA III-IV)
	Pregnancy
BMI 26–35 kg/m^2^	Malignant disease in the past 5 years
Age 30 to 65 years	History of major abdominal operation
	Permanent glucocorticoid or other immunosuppressive therapy
	Pituitary disease/Morbus Addison
	Renal failure (glomerular filtration rate < 45 ml/min)
	Proof of any liver cirrhosis above grade A according to the Child-Pugh-classification
	Expected lack of compliance or inability to informed consent

### Recruitment and timelines

Patients will be recruited by the Study Center of the German Surgical Society (SDGC) at the University of Heidelberg, Department of General, Visceral and Transplant Surgery in cooperation with the Department of Medicine I (Endocrinology) and Clinical Chemistry of the University of Heidelberg and correspondingly at the participating centers. Recruitment is planned to last 12 months. The first patient in to last patient out will be 120 months, and the proposed duration of the entire trial is 132 months.

### Randomization

Randomization will be performed after assessment of the inclusion and exclusion criteria and informed consent of the patient to participate in the study. Randomization will be performed at a ratio of 1:1 for the two treatment groups, using an online randomization tool (http://www.randomizer.at) which enables block randomization and stratification for each center. Four hundred patients will be recruited according to the sample size calculation in order to prevent random error and achieve sufficient strength for hypothesis testing of the primary endpoint.

### Interventions

#### Experimental group

A laparoscopic RYGB will be performed no later than 2 months after allocation to the experimental group. As part of the standardized preoperative assessment, a gastroscopy, laboratory tests, ultrasound of the abdomen, cardiological and endocrinological assessment will be performed.

**Figure 1:**
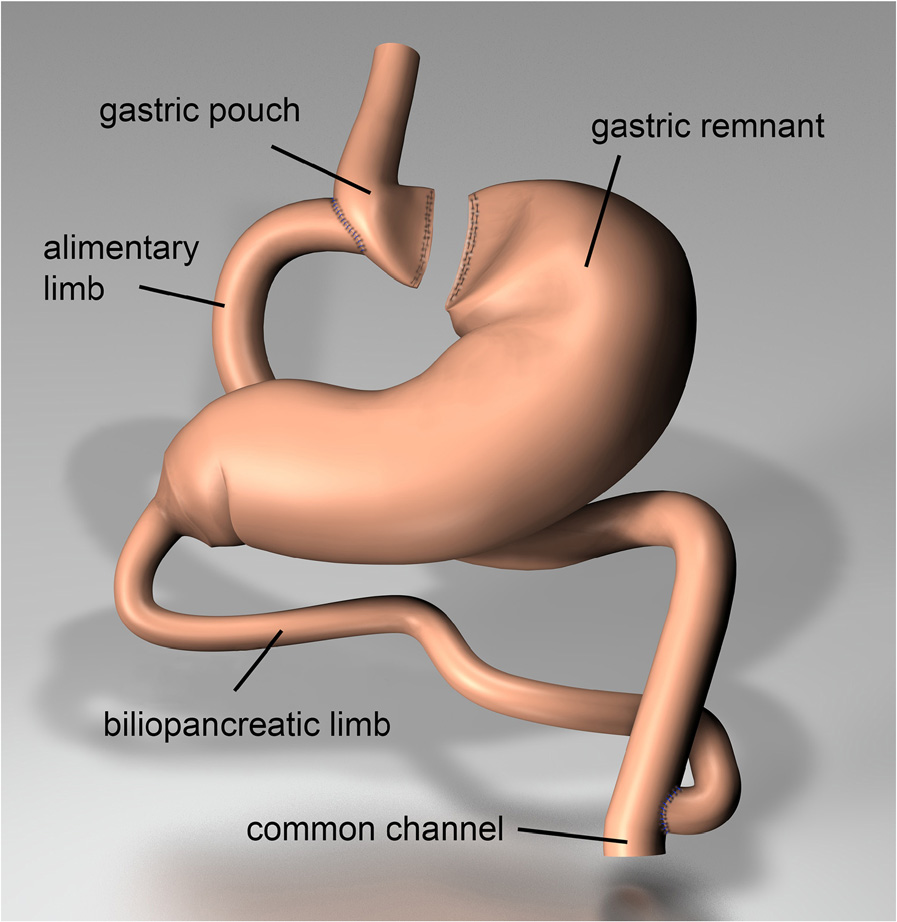
Visualization of the gastric bypass post surgery.

The operation technique is as follows: The patient is positioned in the supine position 30° (reverse Trendelenburg) after a regular fasting time of 12 h. A single-shot of antibiotic prophylaxis is given (according to local standards of participating centers). Then a 15 mmHg pneumoperitoneum through the left upper abdomen is created. Next, five trocars are placed in the upper abdomen. The liver retractor is then positioned in the right upper abdomen. Initially, the gastroesophageal junction is identified. Then the stomach is transsected by the use of linear staplers to build a pouch of 4–6 cm height and 14–16 mm width. The omentum majus can be transected depending on the individual anatomy. At 70 cm from the ligament of Treitz, an end-to-side gastroenterostomy is performed using either a linear or circular stapler. To reconstruct the passage, a side-to-side jejuno-jejunostomy 150 cm from the gastrojejunostomy is performed (Figure 1). The proximal anastomosis is then checked for leaks by methylene blue.

After the operation, patients are hospitalized for approximately 5 days. Diabetic and hypertensive medication are paused and re-administered if needed according to the endocrinologist’s decision. After dismissal, standardized medical therapy of T2DM is administered according to the latest S3 Guidelines of the German Diabetes Association [8,9].

#### Control group

Standardized medical therapy of T2DM and its co-morbidities are defined as the use of the latest S3 Guidelines by the German Diabetes Association [8,9].

#### Study visits

**Figure 2:**
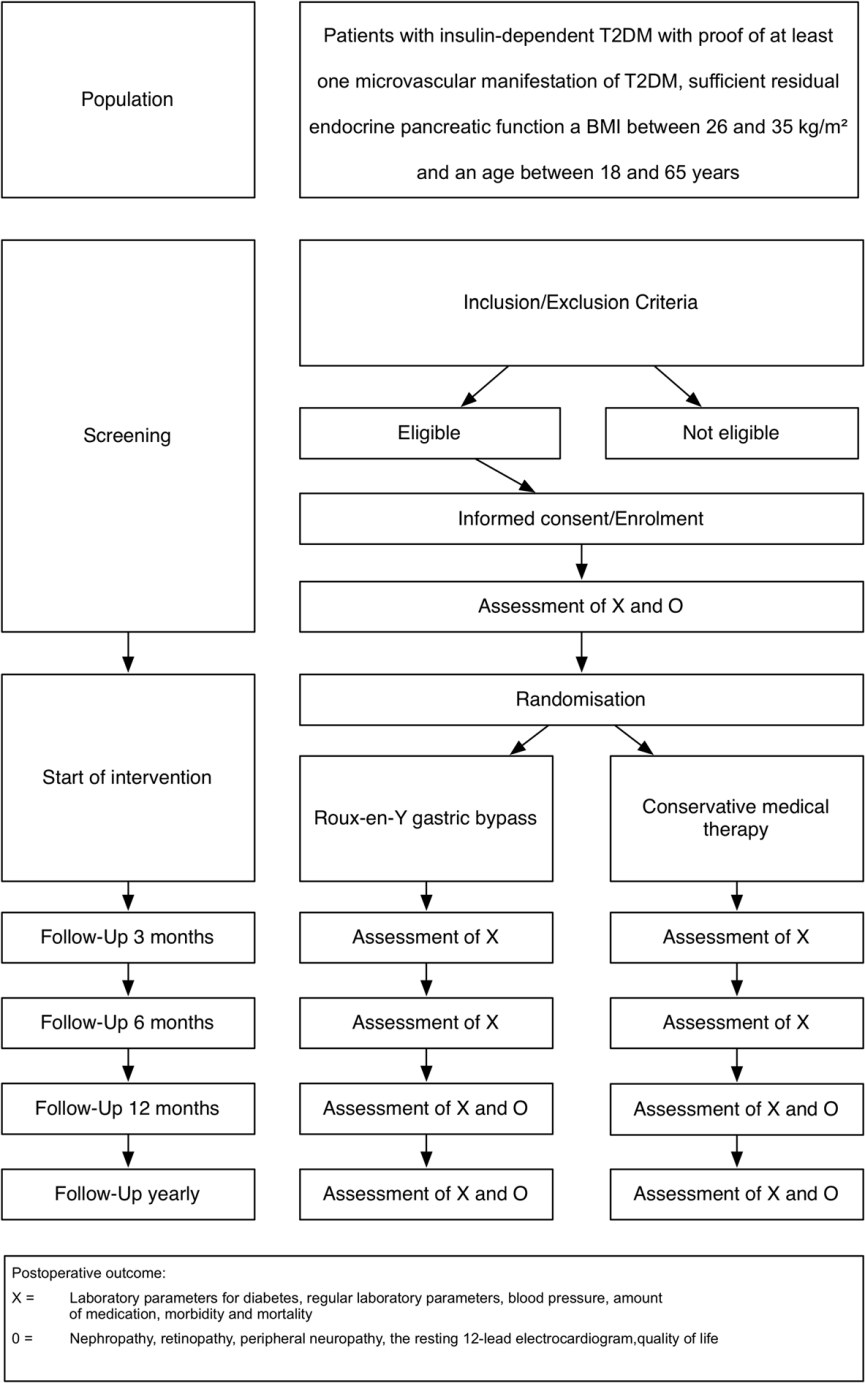
Study flow for the duration of the entire trial.

From screening to last control 8 years after randomization there will be 12 study visits assessing laboratory parameters for diabetes, regular laboratory parameters, blood pressure, resting 12-lead electrocardiogram amount of medication, morbidity and mortality, especially nephropathy, retinopathy, peripheral neuropathy, and quality of life (Figure 2).

### Risk-benefit ratio

RYGB is an established method in bariatric surgery, with low mortality rates of 0.16% to 0.5% in experienced hands [20,28]. Morbidity rates for all-cause complications range between 2% and 10% [20]. Long-term risk for secondary disease due to malabsorption (deficiency in vitamins, foliate, or iron balance) are known and are managed by administering life-long vitamin supplementation [29]. Another possible side effect of RYGB is the dumping syndrome. The risk of surgery for non-severely obese diabetic patients is difficult to assess because there are hardly any data. Due to the fact that patients are not as obese as in bariatric surgery, both reduced morbidity and reduced mortality rates are expected. In recent studies involving non-severely obese patients suffering T2DM, the postoperative mortality rate was found to be 0%, and only 1.2% if the operated patients needed revision due to postoperative complications [19,25,27,30].

### Outcome

The chosen primary and secondary endpoints cover outcomes from different perspectives (for example, patient, surgeon, healthcare system), allowing for a comprehensive evaluation of the surgical treatment of T2DM, especially as to whether long-term T2DM-associated morbidity and mortality can be diminished and which other factors, apart from glycemic control, are dominant.

#### Primary endpoint

The primary endpoint is the time from randomization to one of the composite events, including death from cardiovascular causes, non-fatal myocardial infarction, coronary artery bypass grafting, percutaneous coronary intervention, non-fatal stroke, amputation, and surgery for peripheral atherosclerotic artery disease during the follow-up of at least 8 years according to the Steno 2 study. End-organ damage is also specified according to the Steno2 study from Gaede *et al.*[31,32]. Together with the expected accrual period of 2 years, this amounts to a maximum follow-up period of 10 years. If none of the above events are observed within the follow-up period, the date of the last follow-up is censored.

#### Secondary endpoints

Secondary endpoints include the time to death from any cause and the time to each component of the primary endpoint. In addition, laboratory parameters for T2DM (HbA1c, C-peptide and glucagon stimulated C-peptide, fasting glucose, fasting insulin, oral glucose tolerance test), regular laboratory parameters (that is, lipid profiles, serum creatinine, creatinine clearance) as well as vitamins and micronutrients (vitamin B6, B12, folate acid, and iron status) are assessed. Additional parameters include the amount of medication (insulin dosage, metformin, antihypertensive therapy, and so on), the assessment of nephropathy (urine albumin excretion 24-h urine sample), retinopathy (according to the EURODIAB six-level grading scale), peripheral neuropathy (assessed by biothesiometry of the big toe in both feet and questionnaire), healthcare cost and quality of life. Parameters are assessed at 0, 3, 6, 12 months, and then yearly after surgery (Figure 2).

### Data management

All protocol-required information collected during the trial is entered in the case report form (CRF) by the investigator or a designated representative at the respective participating center. Investigators are expected to complete documentation as soon as possible after the information is collected, preferably on the same day that a trial participant is seen for an examination, treatment, or any other trial procedure.

The completed CRF must be reviewed and signed by the investigator named in the trial protocol or by a designated sub-investigator. After keeping a copy at the trial center, the original CRF is sent to the University of Heidelberg for data entry. Double data entry is carried out to ensure the correct transfer of data from the CRF to the database. Completeness, validity, and plausibility of data are examined by validating programs that thereby generate queries which need to be clarified by the investigator. At the end of the trial, the principle investigator will retain the original CRFs. All data are managed and analyzed by the Study Center of the German Surgical Society (SDGC) at the University of Heidelberg in cooperation with R&P GmbH (Burscheid, Germany) as contract research organization.

### Safety evaluation and reporting of adverse events

During and following a patient’s participation in the trial, the investigator ensures that adequate medical care is provided. The patient receives adequate treatment in any clinical situation, including emergencies, and outcome of the patient must be controlled. A serious adverse event is an event that results in death, is immediately life-threatening, requires or prolongs hospitalization, results in persistent or significant disability or incapacity, or requires re-operation. From the day the patient has signed informed consent until the regular end of the trial, or until premature withdrawal of the patient, all serious adverse events are documented and reported to the principal investigator within 24 h.

### Statistical methods

#### Sample size

The study, with seven components as used in the Steno 2 study [31,32], is designed to demonstrate the superiority of the experimental group to the control group with respect to the hazard ratio for a composite event. The Steno2 study was used to estimate the rate of cardiovascular events we have to expect after 8 years of medical therapy. A reduction of diabetes-associated morbidity from 20%, as reported in the Steno 2 study for intensified conservative T2DM treatment, to 10% for surgical treatment will be defined as success and used for sample size calculation. Let *p*_S_ and *p*_C_ denote the cumulative incidence of one or more components of the composite endpoint for RYGB and standardized medical treatment, respectively. For exponentially distributed event times, event rates of *p*_S_ = 0.10 and *p*_C_ = 0.20 after 8 years correspond to a hazard ratio of 0.472 in favor of surgical treatment [31]. At the two-sided significance level of *α* = 5%, a total number of 56 events is required to detect this effect with a power of 80%. Assuming again exponentially distributed event times, an accrual period of 2 years and a minimal follow-up time of 8 years, the required number of patients to achieve this number of events is given by 336 in total, that is, 168 patients per treatment arm. Assuming a loss to follow-up rate of 20%, 200 patients should be randomly assigned to each intervention group (400 in total). This ensures sufficient power for the intention to treat analysis (ITT) and the complete cases per protocol (PP) analysis.

#### Analysis of the primary endpoint

Each patient’s allocation to the different analysis populations (full analysis set according to the ITT principle, PP analysis set, safety analysis set) will be defined prior to the analysis. The allocation will be documented in the statistical analysis plan prior to database closure. During the data review, deviations from the protocol will be classified as minor or major. Major deviations from the protocol will lead to the exclusion of a patient from the PP analysis set.

The primary analysis will be performed using a Cox proportional hazards regression (*α* = 5%, two-sided) with covariates treatment group, age, duration of T2DM, and a stratification for each center. Confirmatory analysis will be based on the ITT population, with ICA-r imputation for missing values [33].

#### Secondary analyses

A standard sensitivity analysis will be performed using the PP analysis set. A further secondary analysis will be based on a prioritized set of time-to-event variables, ranging from death from any cause (highest priority), stroke, myocardial infarction, amputation, coronary artery bypass grafting, percutaneous coronary intervention, to peripheral atherosclerotic artery disease (lowest priority). Potential treatment benefit will be assessed by the so-called proportion in favor of treatment [34], which is a multivariate permutation test based on pair-wise comparisons of patients. The outcome of Patient X will be said to be better than Patient Y if a decision can be made based on pair-wise comparisons of the components of the prioritized outcome in decreasing order. Surgical treatment with standardized medical treatment will be said to be better than exclusive standardized medical treatment if the aggregate proportion in favor of treatment is above 97.5 percentile of the proportions obtained via simulation under the assumption of the null hypothesis (that is, by permutation of the group membership of the patients).

All secondary variables will be analyzed descriptively by tabulation of the measures of the empirical distributions (means, standard deviations, quartiles). For binary outcomes, relative frequencies will be reported. Descriptive *P* values of the corresponding group comparisons and associated 95% confidence intervals will be given.

#### Safety measures

Safety analysis includes frequency of serious adverse events and complications. Homogeneity of the study arms will be described by comparing demographic data and baseline values.

### Withdrawals and stopping guidelines

Participants are free to withdraw from the trial at any time without explanation. Insufficient recruiting of participants will lead to premature closure, as will exceptionally frequently occurring adverse events due to surgical interventions.

### Data safety monitoring board

An independent Data and Safety Monitoring Board (DSMB) will assess the reports of serious adverse events and will inform the trial management about relevant imbalances between the groups. The DSMB will meet at least twice: once after randomization of one-third of patients, the second time after randomization of two-thirds of the patients.

### Trial organization and administration

#### Funding

The study is funded by the Manfred Lautenschläger Foundation, Gaiberg, Germany and Covidien Services Europe Ltd., Dublin, Ireland.

#### Monitoring

Clinical monitoring will be performed by R&P GmbH (Burscheid, Germany), the contract research organization. Monitoring procedures will be adapted to the study-specific risks for patients and to the guidelines of Good Clinical Practice of the International Conference on Harmonisation of Technical Requirements for Registration of Pharmaceuticals for Human Use (ICH-GCP, E6) to ensure patient safety and integrity of the clinical data, for example, primary endpoint in adherence to the study protocol. Before starting, all participating centers will be personally trained and introduced to all study-specific procedures during a study initiation meeting where the surgical and medical standards are depicted. In addition there will be one video of the standardized operation as an example for each surgeon at the centers. In addition surgeons must have performed at least 20 laparoscopic RYGB to provide sufficient surgical expertise. Regular visits of the participating centers will be performed to check whether the operation is performed as demanded. These visits are planned at all sites depending on the recruitment rate and quality of the data (approximately one visit per site and year). The investigator must allow the monitor to look at all essential documents and must provide support at all times to the monitor. Clinical source data verification (SDV) is planned for all clinical items. The extent of further SDV and/or the frequency of monitoring visits will be adapted for individual centers in case of insufficient quality of data or if common protocol violations are observed. In addition to standard operating procedures, all procedures will be predefined in a study-specific monitoring manual.

### Ethical considerations

The surgical procedure examined in this trial is well established and in current daily use. T2DM is associated with high long-term morbidity and mortality [31]. In obese patients, bariatric surgery showed a remission of T2DM in 42% to 78% of patients [17-19]. In obese patients, mortality of RYGB was about 0.16% to 0.5%, and although the operative risk in obese patients is described, mortality and morbidity rates are expected to be lower in non-severely obese patients [20,28]. Respectively, the operative risk is ethically justifiable considering long-term morbidity and mortality. By comparing conventional medical care to surgical treatment point-by-point, differences in the development of T2DM-associated symptoms are possible. Each individual patient in the intervention group justifiably has the potential to end further insulin treatment for T2DM and thus possibly avoid T2DM-associated co-morbidities. Each patient in the control group will receive standardized medical treatment according to the latest S3 guidelines and thus have no handicap by participating in the study. Participants in the control group will be treated by use of the latest S3 Guidelines set forth by the German Diabetes Association [9]. If the study confirms evidence in improving diabetic symptoms in the experimental group and consequently health-insurance funds refund costs for surgical treatment of T2DM, patients randomized within the medical treatment group will be able to switch to the surgery group. Postoperatively, patients will be examined every year for possible side-effects of the surgery, including an assessment of their vitamin and nutritional status.

#### Good clinical practice

The trial is planned and will be conducted and analyzed according to all relevant national and international rules and regulations (ICH-GCP, Declaration of Helsinki 2008).

#### Registration

The trial protocol has been registered with the German Clinical Trials Register (DRKS) under the registration number DRKS00004550.

## Discussion

Surgical treatment of T2DM is a subject that has been discussed in-depth in recent literature [18,19,35-37]. T2DM remission was observed as occurring before substantial weight loss [18,38]. However, the mechanisms leading to T2DM remission after surgery have not yet been fully understood. Potential explanations include the changed expressions of gastrointestinal hormones, which result in an improved insulin resistance level, and improved excretion of insulin from the beta-cells in the pancreas [39-41]. Currently, however, there is no single satisfactory explanation for the metabolic mechanisms that lead to T2DM remission after bariatric surgery [42]. Nevertheless, T2DM remission was observed in a high proportion of obese patients (42-78%) after bariatric surgery [17-19]. Thus, bariatric surgery is, to some extent, discussed as being part of the therapy regimen for T2DM which may lead to a paradigm shift in the treatment of T2DM, a disease with high prevalence, severe associated co-morbidities as well as being a huge burden on public health [35].

Since it is known that good glycemic control decreases long-term morbidity, a substantial reduction of mortality and morbidity can be expected [6,10]. Several randomized controlled trials (RCTs) have focused on diabetes remission, assessed mostly by HbA1c and fasting glucose, showing a remission of T2DM [21,26,27]. However, diabetes-associated morbidity and mortality were not the subjects of those studies because the follow-up period of 12 to 24 months was too short to assess them as endpoints. These studies have proven that the successful implementation of bariatric surgery for the treatment of non-severely obese patients suffering from T2DM is safe and feasible. Since trials on the long-term effects of bariatric surgery on T2DM in a multicenter setting are not available, the present study was designed not to use HbA1c as a surrogate parameter, but as time-to-event of diabetes-associated mortality or morbidity with an appropriate follow-up of 8 years.

The goal of the study is to assess whether RYGB including standardized medical treatment is superior to exclusive standardized medical treatment of T2DM. Both treatment groups will receive standardized medical treatment according to the latest German S3 guidelines. The endocrinologists at the participating centers agreed on the standardized medical treatment taking the results of the ACCORD study into account [43]. Aggressive insulin dosage will not be performed in any group. Patients receiving RYGB will need close monitoring of dyslipidemia, hypertension, and hypoglycemia and corresponding adaption of the prescribed treatment postoperatively. To ensure this an additional follow-up is scheduled for these patients at 1 month after RYGB.

Sample size calculation and the primary endpoint were based on the Steno 2 study by Gaede *et al.*, who showed a reduction from 30% to 20% of diabetes-associated mortality and morbidity in an intensified medical treatment arm [31]. A further reduction of diabetes-associated morbidity to 10% for surgical treatment was defined as success and used for sample size calculation. To be able to have enough macrovascular complications inclusion and exclusion criteria, for example, microvascular manifestation of T2DM, were adapted to the Steno 2 study to allow for a similar patient population resulting in a study population with high risk to develop macrovascular complications. Although microvascular and macrovascular complications do have different pathogenesis we could use the data from the Steno 2 study to estimate the rate of macrovascular complications in patients with T2DM and microvascular complications at time of randomization. This allows the achievement of sufficient statistical power with a relatively small sample size.

Macrovascular diseases are assessed as time from randomization to one of the components of the primary endpoint including death from cardiovascular causes, non-fatal myocardial infarction, coronaryartery bypass grafting, percutaneous coronary intervention, non-fatal stroke, amputation, and surgery for peripheral atherosclerotic artery disease. Microvascular diseases will be assessed by corresponding laboratory testing; that is, nephropathy (urine albumin excretion 24-h urine sample), retinopathy (according to the EURODIAB six-level grading scale), and peripheral neuropathy (assessed by biothesiometry of the big toe in both feet plus questionnaire).

It is not known how long the effects of surgery on diabetes remission or diabetes-associated co-morbidities last, and if or when T2DM will again require intensified medical treatment. First publications show that in a high proportion of patients the effect is long-lasting. Jimenez *et al.* assessed 153 obese patients after a mean follow-up of 35 months. In 75.2% of patients, remission of T2DM was achieved at 12 months, and in 12.1% of the patients T2DM recurred [44]. Arterburn *et al.* found in 4,434 obese patients having had gastric bypass in a multisite study a median duration of remission of T2DM of 8.3 years [45]. In a further analysis of T2DM incidence, Carlsson *et al*. analyzed 1,658 patients who received bariatric surgery *versus* 1,771 matched obese controls from the SOS study. They found a statistically significant difference namely, that patients who received bariatric surgery were less likely to develop T2DM. The incidence in the control group was 28.4 cases per 1,000 person-years and 6.8 cases per 1,000 person-years in the surgical group [46]. It remains to be discussed whether the benefits of bariatric surgery on T2DM outweighs the risk of the operation. Conservative therapy, however, also has a mortality rate. It was shown by Sjöström *et al*. in an SOS trial involving 4,047 patients receiving bariatric surgery compared to 2,037 matched controls receiving conservative treatment that bariatric surgery reduces mortality in obese patients significantly, with a hazard ratio of 0.76 [47].

Adjustable gastric banding, RYGB, laparoscopic gastric sleeve resection, biliopancreatic diversion, and duodenojejunal bypass have been used for the control of T2DM in non-severely obese patients. All of them show positive effects in the amelioration of T2DM, while gastric banding seems to be the least and biliopancreatic diversion the most effective treatment [17,27,48-50]. In this study RYGB was chosen as the operative technique since it seemed to have the best ratio of risk and benefit in terms of T2DM remission and postoperative mortality and morbidity, for example, severe malabsorption resulting in vitamin and nutritive deficiencies [20,27].

In addition to reduced mortality and morbidity, reduced costs can be expected, as assessed by Makary *et al*., who analyzed 2,235 obese patients with T2DM who underwent bariatric surgery. Compared to preoperative annual healthcare costs of $6,376 in the first year after surgery, costs for T2DM treatment increased by 9.7% but decreased in the third year by 70.5%. Makary *et al*. concluded that even with the additional cost of the operation, overall healthcareexpenditure for patients who received bariatric surgery were lower [51].

If the present study is successful, it will contribute to a possible paradigm shift in T2DM treatment, which will be the implementation of surgery for the treatment of T2DM. This randomized controlled multicentre trial will assess - for the first time in Europe and in a multicenter setting - whether RYGB is an efficient method to prevent long-term complications in non-severely obese patients with T2DM, and whether it can be used as an alternative in the primary care of T2DM, thereby potentially leading to less strokes or cardiovascular death and thus preventing well-known long-term morbidity and mortality in T2DM patients.

## Trial status

Recruitment will begin in Q2 2013.

## Abbreviations

BMI: Body mass index; RYGB: Roux-en-Y gastric bypass; T2DM: Type 2 diabetes mellitus.

## Competing interests

The authors declared that they have no competing interest.

## Authors’ contributions

All authors contributed to the design of the present study and revised the manuscript critically. All authors read and approved the final manuscript.
